# Development of Maximum Residual Stress Prediction Technique for Shot-Peened Specimen Using Rayleigh Wave Dispersion Data Based on Convolutional Neural Network

**DOI:** 10.3390/ma16237406

**Published:** 2023-11-28

**Authors:** Yeong-Won Choi, Taek-Gyu Lee, Yun-Taek Yeom, Sung-Duk Kwon, Hun-Hee Kim, Kee-Young Lee, Hak-Joon Kim, Sung-Jin Song

**Affiliations:** 1 School of Mechanical Engineering, Sungkyunkwan University, Suwon 16419, Republic of Korea; 0won@skku.edu (Y.-W.C.); hjkim21c@skku.edu (H.-J.K.); sjsong@skku.edu (S.-J.S.); 2 Doosan Heavy Industries and Construction Co., Ltd., Changwon 51711, Republic of Korea; taekgyu.lee@doosan.com (T.-G.L.); hunhee1.kim@doosan.com (H.-H.K.); 3 Department of Smart Mechanical Components and Materials, Dongyang University, Yeongju 36040, Republic of Korea; 4 Department of Physics, Andong University, Andong 36729, Republic of Korea; sdkwon@anu.ac.kr; 5 KPC Metal Co., Ltd., Gyeongsan 38412, Republic of Korea; kylee@kpccorp.co.kr

**Keywords:** shot peened, residual stress, Rayleigh wave, convolutional neural network, Inconel 718

## Abstract

Shot peening is a surface treatment process that improves the fatigue life of a material and suppresses cracks by generating residual stress on the surface. The injected small shots create a compressive residual stress layer on the material’s surface. Maximum compressive residual stress occurs at a certain depth, and tensile residual stress gradually occurs as the depth increases. This process is primarily used for nickel-based superalloy steel materials in certain environments, such as the aerospace industry and nuclear power fields. To prevent such a severe accident due to the high-temperature and high-pressure environment, evaluating the residual stress of shot-peened materials is essential in evaluating the soundness of the material. Representative methods for evaluating residual stress include perforation strain gauge analysis, X-ray diffraction (XRD), and ultrasonic testing. Among them, ultrasonic testing is a representative, non-destructive evaluation method, and residual stress can be estimated using a Rayleigh wave. Therefore, in this study, the maximum compressive residual stress value of the peened Inconel 718 specimen was predicted using a prediction convolutional neural network (CNN) based on the relationship between Rayleigh wave dispersion and stress distribution on the specimen. By analyzing the residual stress distribution in the depth direction generated in the model from various studies in the literature, 173 residual stress distributions were generated using the Gaussian function and factorial design approach. The distribution generated using the relationship was converted into 173 Rayleigh wave dispersion data to be used as a database for the CNN model. The CNN model was learned through this database, and performance was verified using validation data. The adopted Rayleigh wave dispersion and convolutional neural network procedures demonstrate the ability to predict the maximum compressive residual stress in the peened specimen.

## 1. Introduction

Material surfaces are in direct contact with the environment and frequently damaged due to corrosion and fatigue loads. The mechanical properties of material surfaces are often improved using surface treatment processes. One of the most popular processes in the industry is the inexpensive and straightforward shot peening process. This process is a cold work surface process, and it uses small spherical sights made of steel and ceramic. A shot injected at high velocity into the surface of a specimen causes compressive stresses on that surface, with maximum compressive residual stresses occurring at a certain depth. After the maximum compressive residual stress to the equilibrium of the material, the compressive residual stress gradually decreases and the tensile residual stress occurs. The compressive residual stress improves the fatigue life of the material. This slows down the crack propagation and requires a certain amount of compressive residual stress from the surface to a certain depth to suppress the cracks [1,2]. Therefore, the evaluation of compressive residual stress is essential in evaluating the integrity of the peened material, and it is primarily evaluated using maximum compressive residual stress and surface compressive residual stress [3].

Zhang et al. [4] performed shot peening on three different materials and found a linear relationship between the maximum depth of the residual compressive stress field after shot peening and the shot peening strength of the material. Sahaya et al. [5] developed an oil jet peening process to form compressive residual stress in metal materials, and they used surface and maximum compressive residual stress to verify the performance. Farrahi et al. [6] concluded in their experiments that improving fatigue life could assign maximum compressive residual stress and the depth of the plastically deformed layer. Wang et al. [7] concluded that a metal’s yield strength and ultimate tensile strength can be calculated through the compressive residual stress and maximum compressive residual stress on the surface of the shot-peened test specimen in the experiment. Likewise, using the maximum compressive residual stress is effective in evaluating the residual stress of peened test specimens, and it is mainly used in evaluating mechanical properties [6,7].

This process of enhancing mechanical properties has been primarily used for nickel-based superalloy steel materials in certain environments, such as the aerospace industry and nuclear power. Nickel-based superalloy steels, such as Inconel and Hastelloy, and titanium alloys are typical of these materials, which retain their mechanical properties at high temperatures. In particular, research on the application of shot peening to Inconel 718, a nickel-based alloy, has been well studied [8,9,10], and it is a material used in various peening studies in the industrial field [11].

Methods for measuring the residual stress generated on an object’s surface are divided into destructive and non-destructive methods. Strain gauge analysis is a representative destructive method, and it has high accuracy, but it has the disadvantage of causing damage to the test material. Non-destructive methods for inspecting and evaluating specimens without damage include X-ray diffraction (XRD), ultrasonic testing, and eddy current testing. Among them, ultrasonic testing is used throughout the industry for material property evaluation. Bulk waves can generally detect internal defects and evaluate mechanical properties, but it is difficult to evaluate the surface properties [12]. 

On the other hand, a surface wave called a Rayleigh wave is primarily used to evaluate the material’s surface properties. The surface wave propagates along a material surface, and energy concentrates on the material surface. 

Many studies have been conducted on defect detection and material surface property evaluation using these characteristics. Kim et al. [13] performed surface defect detection of metal materials using a Rayleigh wave and derived a correlation between the wave’s amplitude and the defect size. M. Duquennoy et al. [14] tested this using the relative variation of the Rayleigh wave time of flight to evaluate the stress distribution on the surface generated during tooling. Kwon et al. [15] concluded that quantitative evaluation of the bonding of ceramic-coated metal materials was possible using a Rayleigh wave. Yeom et al. [16] concluded that the Rayleigh wave can be used to evaluate the surface hardness of metallic material. Fereydoun Lakestani et al. [17] evaluated the thickness of metallic coatings using Rayleigh wave dispersion. Chi-Won In et al. [18] performed a material property analysis of a concrete surface using a Rayleigh wave. X. Jian et al. [19] presented the characteristics of a Rayleigh wave according to surface cracks.

In addition, research on residual stress measurement using the Rayleigh wave has been conducted actively since Ditri and Hongerholt [20]. Ditri and Hongerholt [20] found a relationship between the stress distribution in the depth direction of the material and the Rayleigh wave’s dispersion. Lee et al. [21] measured the Rayleigh wave dispersion using a minimum reflection method. Trung et al. [22] predicted the residual stress through inverse transformation by using the Rayleigh wave dispersion measured by the method and the relationship found by Ditri and Hongerholt [17]. Choi et al. [23] presented an inverse transformation that utilizes an exponential function to predict residual stresses with a simple calculation. However, the transformation had the limitation that it was essential to set the initial values carefully [23]. 

Artificial neural networks (ANNs) are representative machine learning algorithms widely used to solve various engineering system and complex problems. Research and residual stress prediction using ANNs have been conducted in various fields. 

Sembiring et al. [24] predicted nickel alloy steels’ residual stress and hardness subjected to ultrasonic nanocrystal surface modification (UNSM) treatment using an ANN. Hajializadeh et al. [25] predicted residual stresses in parts produced through direct metal deposition using a modeling approach combining finite element analysis and an ANN. Mathew et al. [26] conducted a study with an ANN and feedforward neural networks to predict residual stresses caused by welding. However, research on predicting the residual stress of peened materials using Rayleigh wave dispersion and ANNs is insufficient.

This study presented a method for predicting the maximum compressive residual stress value in Inconel 718 specimens using a prediction ANN model and the relationship between them. As an essential issue in ANN design is the data set, various studies have investigated the residual stress distribution over the depth of the specimen. In addition, a curve fitting method could accurately consider the investigated residual stress distribution development and residual stress distribution conditions to generate 173 distribution data. The generated data were converted to Rayleigh wave dispersion using Ditri and Hongerholt’s equation [20]. Subsequently, a convolutional neural network (CNN) architecture, a convenient ANN structure for extracting features from the input data, was adopted. For learning, 173 distribution data were divided into 140 training data and 33 validation data.

Finally, to confirm the applicability of the learned CNN architecture, the CNN performance was verified with 33 validation data and dispersion data obtained by converting the measured residual stress data investigated in the literature.

## 2. Theory

### 2.1. Characteristics of Residual Stress Distribution by Shot Peening

**Figure 1:**
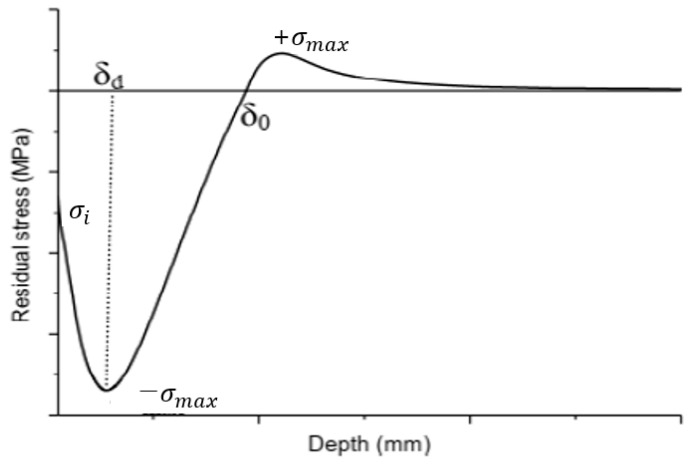
General residual stress distribution using shot peening [20].

Shot peening causes a geometrical change in a material’s surface, and, as a result, compressive residual stress is generated on the material surface. This compressive residual stress reaches its maximum stress at a certain depth and then gradually decreases. Afterwards, tensile residual stress occurs to maintain equilibrium within the material. Figure 1 shows the typical residual stress distribution along the depth of a peened specimen.

As shown in Figure 1, the residual stress generated by shot peening consists of a compressive residual stress ($\sigma_{i})$ at the surface and a maximum compressive residual stress ($\sigma_{max})$ at a specific depth $\left(\delta_{d}\;\sim \;\delta_{0}\right)$. The tensile residual stress for static equilibrium occurs after a compressive residual stress is generated within a specific range $\left(\delta_{d}\;\sim \;\delta_{0}\right)$. 

### 2.2. Relationship between Residual Stress and Rayleigh Wave Dispersion According to Depth

Ditri and Hongerholt [20] studied the relationship between residual stress and changes in surface wave propagation properties using the perturbation theory of surface wave propagating on the surface of a material [20]. According to these studies, the phase shift of the Rayleigh wave, as a type of surface wave propagating on the specimen’s surface, can be calculated using Equation (1).

(1) $$\delta \emptyset =-\frac{\omega }{4P}\int GdV,$$

where δ∅ is the phase shift of the Rayleigh wave, ω is the frequency, V is the volume of the sample, and ∫G is the second-order (λ, μ) and third-order (l, m, n) elastic modulus. This equation predicts the phase shift of the Rayleigh wave during deformation or stress based on the properties of the unstressed specimen (second and third elastic modulus and sound velocity) and Rayleigh wave characteristics, and speed information propagating on the specimen’s surface of the specimen occurs in the specimen [20]. Moreover, the primary assumption is that Rayleigh waves propagate in the $a_{33}$ direction over the length $L_{0}$. The formula for estimating the phase change of a Rayleigh wave is given by Equation (2) [20]:

(2) $$\delta \emptyset^{33}\left(\omega \right)=-\frac{L_{0}\omega }{4P}\int_{0}^{\infty }\alpha_{i}^{∥}F_{i}\left(a_{2},\omega \right)\sigma_{33}\left(a_{2}\right)da_{2},$$

(3) $$F_{i}\left(a_{2},\omega \right)=\omega^{2}\left\{f_{i1}e^{-2\omega K_{s}a_{2}}+f_{i2}e^{-2\omega K_{l}a_{2}}+f_{i3}e^{-\omega \left(K_{l}+K_{s}\right)a_{2}}\right\},\;$$

where $a_{2}$ is the depth direction of the specimen and P is the average power carried per unit width perpendicular to the direction of propagation of the Rayleigh wave during one time. $f_{ij},$ according to the perturbation theory [20], and $i\;\in \left\{1,\ldots ,5\right\}$ and $j\;\in \left\{1,2,3\right\}$, are given as follows:

$$f_{11}={\left(\frac{K_{s}}{V_{0}}\right)}^{2},\;f_{12}={\left(\frac{K_{1}K_{2}}{V_{0}}\right)}^{2},\;f_{13}=-\frac{2K_{S}K_{l}{}_{K_{2}}}{V_{0}^{2}};$$

$$f_{21}=f_{11};\;f_{22}=K_{4}^{2}f_{21},\;f_{23}=-2K_{4}f_{21};$$

$$f_{31}=-2f_{11}\;;\;f_{32}=\frac{K_{l}K_{2}K_{4}f_{31}}{K_{s}}\;,\;f_{33}=-\left[K_{4}+\frac{K_{l}K_{2}}{K_{s}}\right]f_{31};$$

$$f_{41}=\frac{1}{V_{0}^{4}}+K_{s}^{4}\;;\;f_{42}=\frac{K_{2}^{2}}{V_{0}^{4}}+{\left(K_{s}K_{l}K_{4}\right)}^{2}\;,\;f_{43}=-2[\frac{K_{2}}{V_{0}^{4}}+K_{s}^{3}K_{l}K_{4}];$$

$$f_{51}=2f_{11}\;;\;f_{52}=\frac{K_{l}K_{2}K_{4}f_{51}}{K_{s}},\;f_{53}=-\left[\frac{K_{l}K_{4}}{K_{s}}+K_{2}\right]f_{51},$$

where K, according to the perturbation theory [20], can be expressed using the wave velocity, as shown in Equation (4).

(4) $$K_{s}=\sqrt{\frac{1}{V_{0}^{2}}-\frac{1}{V_{s}^{2}}}\;,\;K_{l}=\sqrt{\frac{1}{V_{0}^{2}}-\frac{1}{V_{l}^{2}}}\;,\;K_{2}=\frac{2K_{s}K_{l}}{\left(\frac{1}{V_{0}^{2}}+K_{s}^{2}\right)}\;,\;K_{4}=\frac{2}{\left(1+V_{0}^{2}K_{s}^{2}\right)}$$

$V_{0}$ is the velocity of the Rayleigh wave, and $V_{s}$ and $V_{l}$ are the transverse and longitudinal wave velocities corresponding to the unstressed media, respectively. According to the perturbation theory [20], the constants $\alpha_{i}^{∥}$ in Equation (2) are as follows:

$$\alpha_{1}^{∥}\equiv \frac{1}{\left(3\lambda +2\mu \right)}\{\lambda +2l-\frac{\lambda \left(2\lambda +6\mu +4m\right)}{2\mu }\};$$

$$\alpha_{2}^{∥}\equiv \frac{1}{\left(3\lambda +2\mu \right)}\{\lambda +2l-\frac{\left(\lambda +\mu \right)\left(2\lambda +6\mu +4m\right)}{\mu }\};$$

$$\alpha_{3}^{∥}\equiv \frac{1}{\left(3\lambda +2\mu \right)}\left\{\lambda +2l-\frac{\lambda \left(\lambda +2m-n\right)}{2\mu }\right\};$$

$$\alpha_{4}^{∥}\equiv \frac{1}{\left(3\lambda +2\mu \right)}\left\{3\lambda +2\mu +m-\frac{\lambda \left(2\mu -\frac{n}{2}\right)}{2\mu }\right\};$$

$$\alpha_{5}^{∥}\equiv \frac{1}{\left(3\lambda +2\mu \right)}\left\{\lambda +\mu +m-\frac{\lambda \left(\mu -\frac{n}{2}\right)}{2\mu }\right\}.$$

Equation (2) indicates that the phase change has a linear relationship with the stress distribution in the stressed specimen [20,27]. Based on the experiment in [27], this phase change can be summarized using Equation (5) [20].

(5) $$\frac{∆V}{V_{0}}=\bar{\varepsilon_{33}}-\frac{\delta \emptyset^{33}V_{0}}{\omega L_{0}},$$

where $\bar{\varepsilon_{33}}$ represents the surface strain in the wave’s propagation direction and $\frac{∆V}{V_{0}}$ represents the relative change in the wave’s phase velocity propagating along the surface of the stressed specimen [20]. Therefore, if the residual stress along the depth is known, ∆$\frac{∆V}{V_{0}}$ can be obtained; this $\frac{∆V}{V_{0}}$ can be used to predict the stress by measuring the Rayleigh wave for the unstressed and deformed specimen with deformation.

### 2.3. Convolutional Neural Network (CNN)

A CNN is a deep neural network (DNN) that performs convolutional operations. The CNN structure was announced through Fukushima in 1979 under the term “Neocognitron” and assumed its current form after LeCun et al. proposed a CNN with a backpropagation structure [28,29].

**Figure 2:**
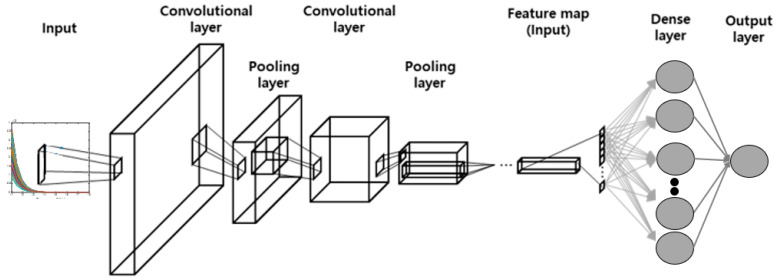
Schematic of the CNN architecture [28].

Like a DNN, a CNN has a structure wherein data enter the input layer, pass through the hidden layer, and emanate from the output layer. Additionally, it uses a convolutional layer and a pooling layer. The convolutional layer uses convolution between filters and data to extract meaningful features from the data. In other words, when the input data are filtered, the data dimension is reduced several times, which has the advantage of reducing the amount of computational data but may cause data loss in some cases [30]. Therefore, a pooling layer is used to retain the characteristics. A technique often used in the pooling layer is max pooling, which reduces the computation required by reducing the dimensionality and extracting salient features. As a result, the convolutional layer and pooling layers are primarily together, and the feature maps of the data are extracted by repeating this structure. Finally, the extracted feature map is combined with the next dense layer to obtain the predicted data values as the output of the CNN architecture. Figure 2 shows the CNN schematically.

## 3. Construction of the Database of Rayleigh Wave Dispersion

In this study, a database was created based on Rayleigh wave dispersion data to estimate the maximum compressive residual stress value of shot-peened Inconel 718 test specimens. First of all, to obtain Rayleigh wave dispersion data, residual stress distribution along depth is required, so XRD data of peened Inconel 718 specimens measured in various studies were used [31,32,33,34,35]. XRD data are residual stress values measured at various depths of the specimen, and in order to convert them into a continuous residual stress function according to depth, each piece of XRD data was modeled as a continuous function using curve fitting. Curve fitting was performed using a Gaussian function consisting of an exponential function to perform the transformation effectively and efficiently from the stress distribution to the Rayleigh wave distribution using Equation (5) [23]. The Gaussian function used in this study is given by Equation (6):

(6) $$y=a*e^{\left[-{\left(\frac{x-b}{c}\right)}^{2}\right]}.$$

where x is the depth of the specimen and y is the estimated residual stress function at a specific x. The distribution of the residual stress expressed by Equation (6) adequately describes the distribution of the residual stress generally generated in industrial fields. To describe this adequately, the ranges of the three variables (a, b, and c) used in Equation (6) should be set appropriately based on the XRD measurements in references [31,32,33,34,35]. Variable a represents the residual stress value of a fitting curve, variable b represents the position of the maximum compressive stress, and variable c represents the variance. The set range is as shown in Equation (7).

(7) $$-1.4*10^{9}\leq a\leq -8*10^{8}\;0.03\leq b\leq 0.07\;0.05\leq c\leq 0.11$$

**Figure 3:**
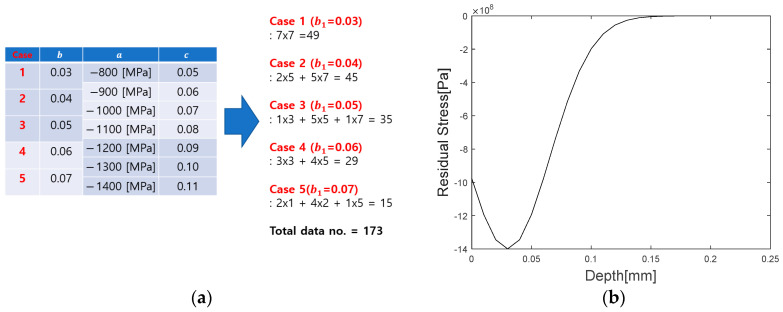
(**a**) Constructed database and (**b**) Specific distribution residual stress data in the configured database.

The ranges of each variable derived from Equation (7) imply the following:

(1) The maximum compressive residual stress of the peened material was set not to exceed the yield strength [36,37,38]. (2) The residual stress generated at a 0 mm depth (surface) of the test specimen was not set more significantly than the maximum compressive residual stress, nor was it set too excessively smaller. Subsequently, cases were classified based on the value of b, and the database was constructed using a factorial design approach [39]. Figure 3 shows the constructed database.

**Figure 4:**
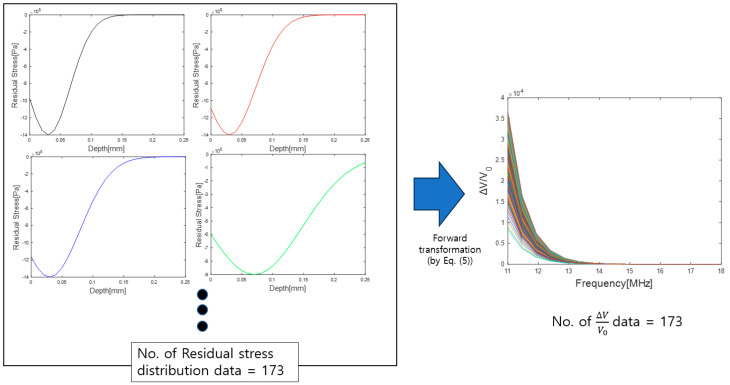
Transformation of the residual stress distribution to Rayleigh wave dispersion data.

Figure 3a shows a database with 173 residual stress distributions created by assuming variables that satisfied the range in Equation (7). Among the variables of the Gaussian function, b, which is the variable that determines the location of the maximum compressive residual stress, was set first. In addition, variable a, which is related to the maximum compressive residual stress value, was set, and, finally, C, which represents the slope of the residual stress distribution, was set. Figure 3b shows one data of the 173 residual stress distribution data created based on classified cases. Figure 4 shows the transformation of 173 residual stress distribution data modeled with a Gaussian function into Rayleigh wave dispersion data ($\frac{∆V}{V_{0}})$.

As shown in Figure 4, Equation (5) was used to convert these data into Rayleigh wave dispersion data for a specific frequency range to generate data for CNN training and input layers. Among the 173 dispersion data points created in this manner, 140 were used as training data, and the remaining 33 were used as test data.

## 4. CNN Architecture

**Figure 5:**
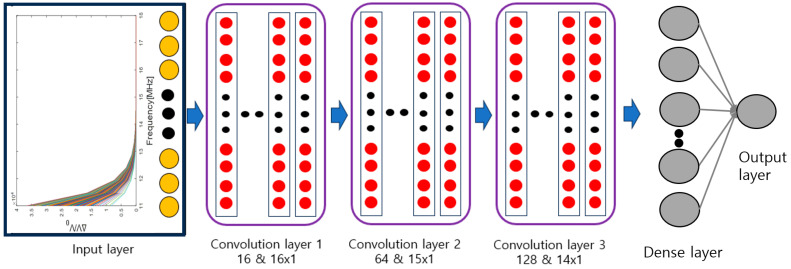
Architecture of CNN.

This study’s CNN architecture was adopted for learning designs using the Jupyter Notebook 3.11.3 from Python and Tensorflow (Google Open Source for Deep Learning). This structure consisted of an input layer, three convolutional layers, a max pooling layer, and one output layer. The number of nodes in the input layer was 16, which was obtained by dividing the range from 11 to 18 MHz in 0.5 MHz steps. The filter sizes in each convolutional layer were 16 × 1, 15 × 1, and 14 × 1. The number of filters was 16, 64, and 128, and the strides were set to 1, 1, and 3, respectively. The activation function used was the rectified linear unit (ReLU), and size of the max pooling size used to extract the features was 2 × 1. Therefore, through the corresponding structure, the size of the final output layer was 1 × 1, and this was the maximum compressive residual stress prediction value for the input value (Rayleigh wave dispersion data) of the input layer. Figure 5 shows a schematic diagram of the input layer and the CNN structure.

## 5. Results and Validation

### 5.1. Performance Evaluation of the CNN Model

**Figure 6:**
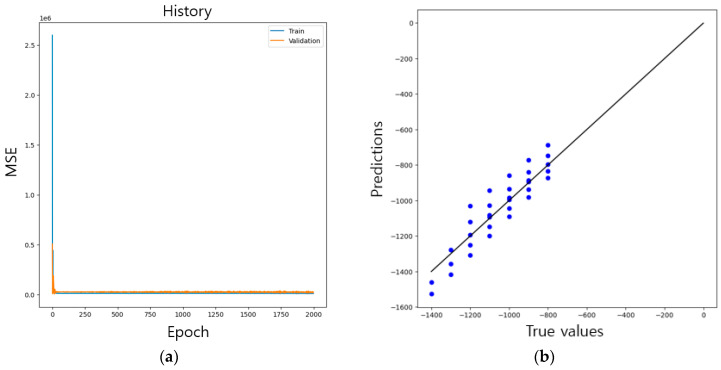
(**a**) Learning curves of CNN and (**b**) scatter plot for evaluating performance.

The performance of the CNN was evaluated using test data after training the neural network using the training data. Figure 6 shows the results of the CNN training and performance evaluation.

Figure 6a shows the learning curve according to the epoch. The loss value decreased as the epochs increased, and the mean squared error (MSE) was used as a function to reduce the loss. Table 1 numerically presents MSE values according to the epoch.

Thus, the network was trained using 140 data points and tested using 33. Figure 6b shows a scatter plot of the performance obtained when inputting 33 test data to a CNN trained with 2000 epochs. In Figure 6b, the solid line represents the ideal case, thus showing the performance with an R-squared value of 79%. Table 2 presents the performance evaluation results of the test data.

Table 2 shows the prediction values obtained using experimental data for the CNN. For the average error of the predicted values for each category, the maximum error was 6.2%, and for the predicted values, it was predicted to be within 15%.

### 5.2. Validation with XRD Fitting Data

**Figure 7:**
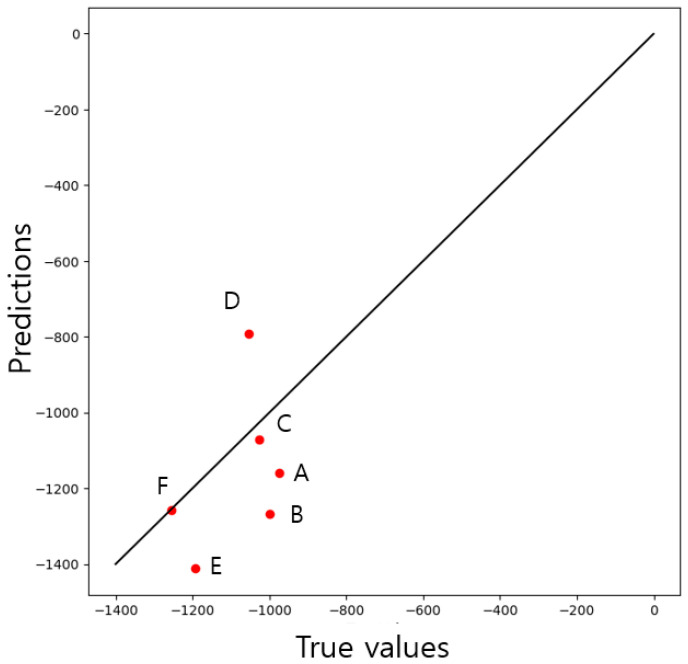
Validation results.

Using the XRD results reported in previous studies [21,31,32,33,34,35], the performance of the trained CNN was further verified. For verification, the same process as the database construction process was applied to the XRD results, and, finally, Rayleigh wave dispersion data were created for the XRD results of each study. Figure 7 shows the further validation results for the learned CNN structure with the Rayleigh wave dispersion data.

The true maximum compressive residual stress values of each of the XRD validation data input to the CNN are 975 (A), 999 (B), 1027 (C), 1055 (D), 1194 (E), and 1256 MPa (F). Figure 7 shows the validation results as a scatter plot. Table 3 shows the predicted values and errors for the validation data.

As shown in Table 3, the predicted values for each maximum compressive residual stress are 1160.1, 1266.9, 1072.1, 792.6, 1410.2, and 1256.3 MPa. In addition, the maximum error is 24.9%, the minimum error is 0.01%, and the average error of the predicted value is 13.64%, which is similar to the performance evaluation results with the test data.

## 6. Discussion

From the performance evaluation using a database of 173 data points created based on XRD reference data [31,32,33,34,35], the coefficient of determination was 79%, and the predicted value was within 15%. However, in the additional validation results after curve fitting to the XRD data of references [21,31,32,33,34,35], the coefficient of determination was negative, and the predicted value was within 25% of the maximum. This is because the C [33] and F [31] data are very close to the true value, and the prediction error of E [32] is within 15%, but the error of other data is significant. In particular, when curve fitting the XRD data of reference [21], variable a satisfied Equation (7) as −1.05 × 10^9^, but variable b (44.9) and variable c (45.9) were not satisfied. In addition, this confirms that A and B have prediction errors of 15% or more, and Table 4 below shows the depth at the maximum stress of the curve fitting value obtained using XRD data from each study.

Table 4 shows the depths at which the maximum stress occurs in data A and B, which are 0.035 and 0.025 mm. When using Equation (5), the higher the frequency of the Rayleigh wave, the more accurate the conversion in the range close to the surface. This is because most of the Rayleigh wave energy is concentrated in a thin region near the surface [40]. Therefore, because the currently used frequency band is about 20 MHz, it is expected that A and B prediction errors can be effectively reduced by raising the frequency band or using a correction coefficient to increase the frequency band.

## 7. Conclusions

In this study, the maximum compressive residual stress was estimated to evaluate the residual stress for peened Inconel 718, and a method for predicting the maximum stress value using Rayleigh wave dispersion and a CNN model is proposed.

A database for CNN model learning was composed of Rayleigh wave dispersion data. To produce the Rayleigh wave dispersion, curve fitting was performed on the XRD stress data from the investigated literature [31,32,33,34,35]. For curve fitting, the Gaussian function was adopted to efficiently perform the transformation using Equation (5), and the range of the variables of the Gaussian function was set by referring to various studies [36,37,38]. Using this range, the depth at which the maximum compressive residual stress occurs in the residual stress distribution was classified on a case-by-case basis, and a total of 173 residual stress distribution data were generated case by case. To create data for the CNN input layer, the 173 residual stress distribution data according to depth were converted to a Rayleigh wave dispersion curve using Equation (5). The database constructed 140 training and 33 test data to estimate the maximum compressive residual stress value.

The CNN structure uses the converted Rayleigh wave dispersion as input to predict the maximum compressive residual stress value. In order to effectively extract the features of the dispersion curve, the CNN is constructed with three convolutional layers and one max pooling layer and then trained.

According to the results of the CNN performance evaluation using the experimental data, the average error of the predicted values was higher than 6.2%, and the predicted value was within 15%. The six residual stress data from the research literature used to design the database were converted to the dispersion data through Equation (5), and the average error of the predicted value obtained by performing additional validation with the dispersion data was 13.64%. If the Rayleigh wave dispersion for the peened specimen is acquired experimentally, it is expected that it can be available to evaluate the residual stress through the experimental data and the prediction CNN model. The approach to estimating the maximum residual stress using a CNN presented in this study is expected to be a valid method for use in industrial settings.

## Figures and Tables

**Table 1:** MSE values by epoch.

Epoch	MSE (Training Data)	MSE (Test Data)
1	1,298,132.25	333,497.37
500	12,858.45	29,176.74
1000	13,305.45	25,051.67
1500	12,199.92	23,150.61
2000	8434.2	14,202.05

**Table 2:** CNN performance results.

True Value of Maximum Compressive Residual Stress	Unit: MPa						
	800	900	1000	1100	1200	1300	1400
Predicted value of maximum compressive residual stress	871.2	938.5	1089	1197.9	1306.9	1415.8	1524.7
Predicted value of minimum compressive residual stress	686.8	772.7	858.5	944.4	1030.2	1278.4	1460
Predicted value of average compressive residual stress	787	885.38	983.76	1082.13	1180.57	1349.98	1492.33
Predicted value of error	−14.2%–+8.9%	−14.2%–+4.2%	−14.2%–+8.9%	−14.2%–+8.9%	−14.2%–+8.9%	−1.7%–+8.9%	−4.1%–+8.9%

**Table 3:** CNN validation results.

Maximum Compressive Residual Stress True Value	Unit: MPa					
	975 (A) [34]	999 (B) [35]	1027 (C) [33]	1055 (D) [21]	1194 (E) [32]	1256 (F) [31]
Predicted value (max)	1160.1	1266.9	1072.1	792.6	1410.2	1256.3
Predicted value error	16.2%	21.2%	4.2%	24.9%	15.34%	0.01%

**Table 4:** Validation results.

XRD Validation Data from References	The Depth of the Maximum Compressive Residual Stress Value from the Surface (Unit: mm)
A [31]	0.035
B [32]	0.025
C [30]	0.04
D [21]	0.045
E [29]	0.046
F [28]	0.057

## Data Availability

The data that support the findings of this study are available from the corresponding author upon reasonable request.
